# Examining the effect of Libet clock stimulus parameters on temporal binding

**DOI:** 10.1007/s00426-021-01546-x

**Published:** 2021-06-12

**Authors:** Bianca E. Ivanof, Devin B. Terhune, David Coyle, Marta Gottero, James W. Moore

**Affiliations:** 1 Department of Psychology, Goldsmiths, University of London, New Cross Road, London, SE14 6NW UK; 2 Department of Computer Science, University College Dublin, Dublin, Ireland

## Abstract

Temporal binding refers to the subjective temporal compression between actions and their outcomes. It is widely used as an implicit measure of sense of agency, that is, the experience of controlling our actions and their consequences. One of the most common measures of temporal binding is the paradigm developed by Haggard, Clark and Kalogeras (2002) based on the Libet clock stimulus. Although widely used, it is not clear how sensitive the temporal binding effect is to the parameters of the clock stimulus. Here, we present five experiments examining the effects of clock speed, number of clock markings and length of the clock hand on binding. Our results show that the magnitude of temporal binding increases with faster clock speeds, whereas clock markings and clock hand length do not significantly influence temporal binding. We discuss the implications of these results.

## Introduction

The sense of agency refers to the feeling of control over actions and their effects (Haggard, 2005). Synofzik et al. (2008) propose a distinction between explicit sense of agency, which represents the higher order explicit judgments/attributions of agency, and implicit sense of agency, which represents the low-level, non-conceptual feeling of control over actions and events. Explicit sense of agency is studied by asking the participant to make a judgment about their own causal efficacy or attribute agency to the self or others. In contrast, implicit sense of agency is examined by measuring perceptual correlates of voluntary action (Moore, 2016).

One widely used implicit measure of sense of agency is temporal (or intentional) binding (Haggard et al., 2002). This measure is based on changes in time perception that accompany voluntary action. Haggard and colleagues found that when we make voluntary (as opposed to involuntary) movements, we perceive our actions as occurring later in time and closer in time to associated outcomes, and action outcomes as earlier in time, closer to associated actions.

Traditionally, temporal binding is measured using the so-called “Libet clock”, introduced by Libet et al. (1983) in an attempt to study subjective timing of events. An analogue clock marked at conventional intervals (5, 10, 15, etc.) was presented on a screen in front of the participants. On the outside perimeter of the clock face, a spot rotated around the clock at a speed of one revolution every 2560 ms. Participants used this clock to estimate the time they became aware of their intentions to act (“W judgments”), the time they performed actions (“M judgments”), and the time they perceived a somatosensory stimulus touch their skin (“S judgments”). Although the Libet clock method is widely used to measure temporal binding (Moore & Obhi, 2012), it is not clear how and why the clock stimulus parameters were set as they were. More importantly, it is unclear whether and to what extent these clock stimulus parameters modulate temporal binding.

Libet clock stimulus parameters are important, because there is a degree of inconsistency in the clock settings that are used across different studies. One such example is inconsistency in clock markings. In Libet’s original study, the clock was marked at conventional intervals (see above), which also contained radial lines equally spaced between these intervals (2.5, 7.5, 12.5, etc.). However, other studies used a rectangular clock with numbers from 1 to 12 equally spaced around the perimeter (Trevena & Miller, 2002), an unnumbered clock (Lau et al., 2006), a clock marked at conventional intervals only (Haggard et al., 2002), or a clock marked at 60 equally spaced positions (1, 2, 3, etc.) (Demanet et al., 2013). Studies also vary in the rotating stimulus that is used (e.g. a red rotating spot—Libet et al., 1983; a black clock hand—Haggard et al., 2002; a cursor—Isham et al., 2011; a black rotating spot—Caspar & Cleeremans, 2015), as well as in the distance between the rotating stimulus and the perimeter of the clock face (e.g. Capozzi et al., 2016; Desantis et al., 2011; Engbert & Wohlschläger, 2006; Moore & Haggard, 2008; Takahata et al., 2012; Wenke et al., 2009).

The use of uniform stimulus parameters across studies is important for replication purposes, particularly if changes in these settings have an effect on timing judgments, which form the basis of the temporal binding effect. This may indeed be the case. For example, Pfister et al. (2021) found that action binding was absent when trials were terminated immediately after the tone effect was presented, compared to trials where the clock hand continued rotating for a variable interval (as is standard practice in the Libet clock paradigm).

Also of relevance to the present investigation is a study by Pockett and Miller (2007). They manipulated seven different factors of the clock method simultaneously to investigate these manipulations’ influence on participants’ M judgments. Some of these factors were, for instance, whether the clock radius was large or small, whether the spot rotated rapidly or slowly, and whether the participants were required to report the start or the end of their key-presses. They found the latter factor to moderately affect participants’ M judgments.

Finally, Danquah et al. (2008) also looked at the influence of manipulations of Libet clock stimuli on basic timing judgments. They investigated if changes to clock rotation speed influenced S judgments. They used a spot marker that rotated at speeds of 1280 ms, 2560 ms or 5120 ms per revolution. Critically, they found that participants’ awareness of the somatosensory stimulus used was less anticipatory (relative to its actual time of occurrence) the faster the clock speed was.

In light of these issues, various attempts have been made to tackle the limitations of the Libet clock and develop additional binding measures. For instance, interval estimation approaches reliably reproduce the critical finding by requiring participants to simply estimate the length of the interval between their self-paced button-press and the ensuing tone (e.g. Humphreys & Buehner, 2009; Moore et al., 2009). Further to this, versions of the Libet task that do not rely on an analogue clock have also been developed. For example, binding effects have been observed when the clock has been replaced by the visual or auditory presentation of letters (Cavazzana et al., 2014; Cornelio Martinez et al., 2018; Muth et al., 2020).

These aforementioned studies draw attention to possible issues with the Libet clock methodology, in particular with respect to the clock stimulus itself. However, no study has systematically investigated the role of the clock stimulus (and its parameter settings) on the expression of the binding effect. This is something the present investigation set out to do.

In light of the inconsistency across studies in Libet clock settings reported above, coupled with preliminary evidence that stimulus parameters might modulate timing estimates (e.g. Danquah et al., 2008; Pockett & Miller, 2007), we conducted a systematic investigation of the impact of Libet clock stimulus parameters on temporal binding. In five experiments, we investigated the effect of changes in clock speed, the number of clock markings and clock hand length. Our aim was to isolate stimulus parameters that influence temporal binding and thereby draw attention to the role of stimulus features on temporal binding.

## Experiment 1

In this experiment, we investigated the influence of clock rotation speed on temporal binding. We have already seen that it might affect basic timing judgements (e.g. Danquah et al., 2008). Following Danquah et al. (2008), we used three different rotation speeds: one revolution every 1280 ms (fast), every 2560 ms (standard rotation speed) and every 5120 ms (slow). For each rotation speed, we ran the standard temporal binding procedure whereby participants are asked to either press a button that, across different blocks of trials, occurs in isolation or is followed by an outcome, or hear a tone generated by the computer. In each of these four conditions, participants were required to estimate the time either the button-press or the tone occurred.

### Methods

#### Participants

40 participants (*M*_age_ = 26.3 years, SD = 8.64, age range 18–61, 15 males) were recruited to the study using existing databases and the Goldsmiths Research Participation Scheme. They were compensated with £7 or 7 course credits for approximately 1hr30mins of experimental time. Insofar as there was uncertainty regarding the anticipated effect size for the expected effect, we opted a priori to include 40 participants in this study, which would give us 80% power (assuming alpha = 0.05; sphericity = 0.8; three levels) to detect effect sizes of *ɳ*_p_^2^ = 0.226 and above for the main effect of our primary independent variable of interest (clock speed). Our final dataset contained 39 participants (*M*_age_ = 26.3 years, SD = 8.75, age range 18–61, 14 males) after one participant was excluded (see “Data analysis”). All participants were right-handed, had normal or corrected-to-normal vision, with no self-reported psychiatric or neurological disorders or substance usage that might interfere with their cognitive performance. They provided written informed consent prior to participation and the experiment was approved by the Department Ethics Committee at Goldsmiths, University of London.

#### Materials

Temporal binding was measured using a Libet clock task programmed in JAVA (version 6; ORACLE, 2011). The clock measured 21 mm in diameter, featured a 9 mm hand and was marked at conventional intervals (5, 10, 15, etc.) in all blocks and conditions. The tone in the baseline and both operant conditions (see below) was presented at 1000 Hz and lasted for 100 ms.

#### Procedure

Participants were presented with three different clock speeds in separate conditions (1280 ms, 2560 ms and 5120 ms per clock revolution). As part of each clock speed condition, participants completed four different binding blocks (operant action, operant tone, baseline action, baseline tone), each containing 30 trials, resulting in 12 blocks (360 trials) per participant. The speed conditions were counterbalanced across participants and the binding blocks were fully randomised within participants.

Participants completed three practice trials (2560 ms speed) prior to each binding block. That resulted in 12 additional trials per participant, which were discarded. Afterwards, they started the main task.

**Fig. 1:**
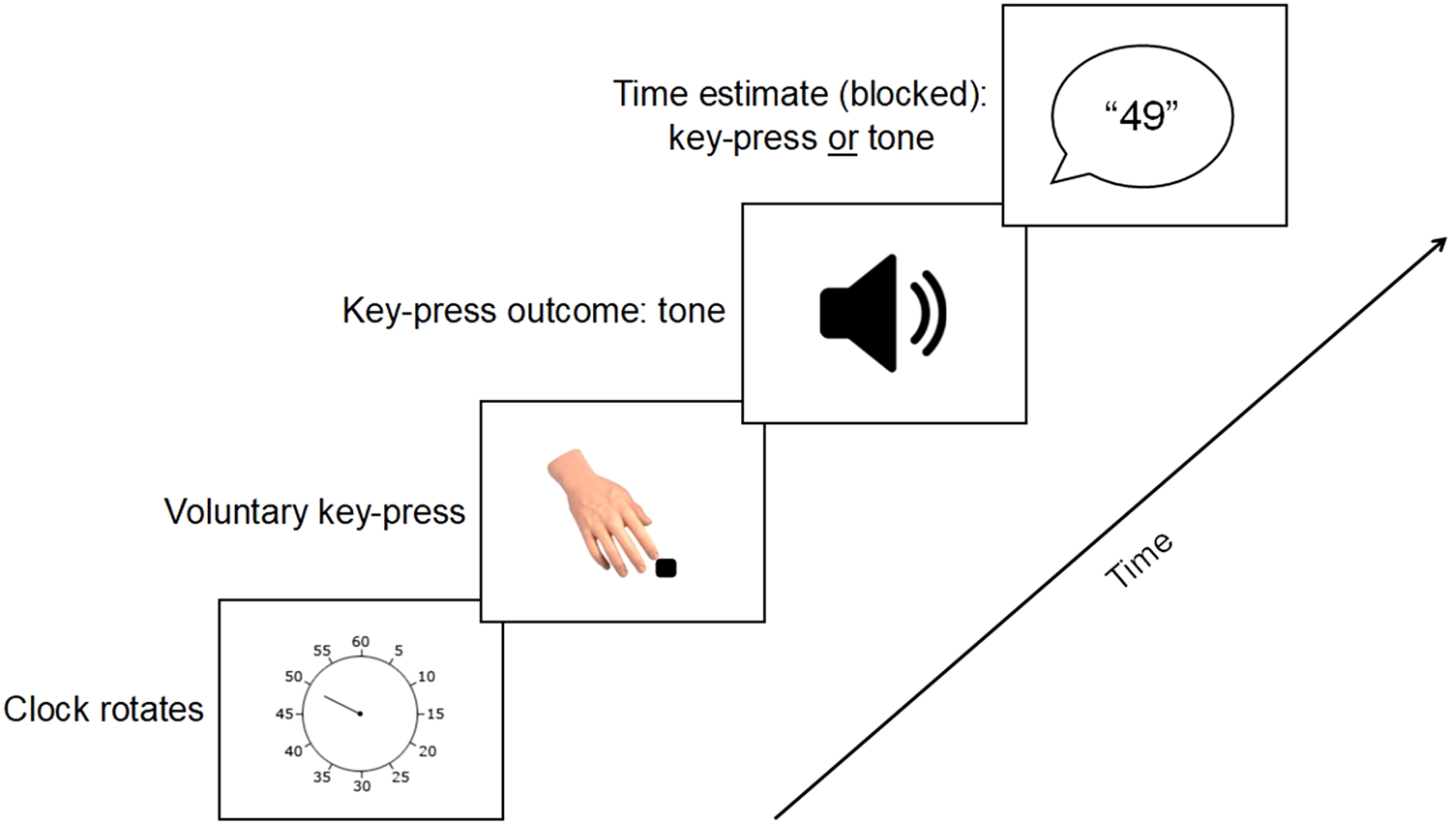
Standard trial structure in operant blocks following Haggard et al. (2002). Participants were required to press a key at their own pace, which was followed by a tone 250 ms later. Depending on the operant block type, they had to report the time of their action or of the action outcome

As can be seen in Fig. 1, in the operant blocks, participants were required to press a pre-specified key whenever they felt the urge to do so. Their key-press was always followed by a tone after a fixed interval of 250 ms. Their task was to estimate the position of the clock hand on the clock face when they pressed the key (operant action block) or heard the tone (operant tone block). Once the clock hand stopped rotating after a random delay following the tone (1000–2500 ms), participants verbally reported the estimated clock time to the experimenter, who inputted the number.

Participants also had to complete two baseline blocks. In the baseline action block, participants pressed the key whenever they felt the urge to do so and subsequently reported the clock time corresponding to their key-press to the experimenter (the clock here stopped rotating after a random time between 1000 and 2500 ms following the action). The experimenter specified to all participants that actions performed in the baseline block would never be followed by an outcome. In the baseline tone block, participants were instructed to pay attention to the location of the clock hand on the clock face when hearing a tone generated by the computer. This tone occurred at a random time between 2500 and 5000 ms after trial onset and participants were asked to report the perceived time of the tone to the experimenter.

Participants sat at a distance of approximately 65 cm from the clock face across all speed conditions (visual angle: approximately 1.8°). We always informed them of any change in clock speed before completing a new condition, given opportunities to rest in-between conditions, and were reminded not to pre-plan their movements and be as accurate as possible when reporting their time estimates to the experimenter.

#### Data analysis

Raw judgment errors were calculated as the perceived time minus the actual time of action or tone onset. This resulted in four raw judgment errors for the four binding blocks. Action binding was computed as the mean operant—mean baseline action judgment error, whereas Tone binding was computed as the mean operant—mean baseline tone judgment error. These measures were computed for each clock speed condition.

We excluded individual trials containing raw judgment error outliers within participants (M ± 2.5 SDs). This criterion resulted in 1.85%, 1.92% and 1.83% trials excluded across all four binding blocks in the 1280 ms, 2560 ms and 5120 ms clock speed condition, respectively. We removed outliers at the group level if a combination of factors indicated univariate or multivariate outliers (Field, 2013; these included skewness and kurtosis values greater than approximately ± 2.000, significant results rendered by the Kolmogorov–Smirnov test, visual inspection of boxplots and histograms).

#### Statistical analyses

All data and statistical analyses were conducted using MATLAB (v. R2012a, MathWorks, Natick, MA) and IBM SPSS Statistics (v. 22 and 23; 2014, 2018). The results of the Kolmogorov–Smirnov test indicated that all but two factors were normally distributed. Closer inspection of the skewness and kurtosis values, histograms and boxplots of the factors in question raised no major issues, so we decided to proceed forward using parametric analyses (which are relatively robust against these minor perturbations in normality; Field, 2013). Thus, we ran a 3 × 2 repeated-measures ANOVA with Clock speed (1280 ms, 2560 ms, 5120 ms) and Event (Action binding, Tone binding) as within-subject factors and mean baseline-corrected judgment error as a dependent variable.

### Results

Participants’ mean action and tone timing scores across all four baseline and operant blocks, as well as their mean binding scores across all three clock speed conditions are shown in Table 1.

**Table 1:** Mean raw and baseline-corrected judgment errors as a function of clock speed

Rotation speed condition (ms/revolution)	Judged event	Mean raw judgment error (ms) (SD)	Mean shift from baseline (ms) (SD)
1280 ms	Action		
	Baseline	− 23.25 (68.30)	36.80 (42.92)
	Operant	13.54 (79.20)	
	Tone		
	Baseline	28.09 (56.65)	− 73.79 (76.52)
	Operant	− 45.69 (100.06)	
2560 ms	Action		
	Baseline	− 7.72 (72.28)	26.62 (35.78)
	Operant	18.90 (84.81)	
	Tone		
	Baseline	16.29 (75.17)	− 60.49 (82.62)
	Operant	− 44.19 (108.28)	
5120 ms	Action		
	Baseline	11.75 (72.61)	35.38 (41.71)
	Operant	47.13 (78.62)	
	Tone		
	Baseline	26.79 (79.97)	− 42.88 (97.44)
	Operant	− 16.09 (124.52)	

**Fig. 2:**
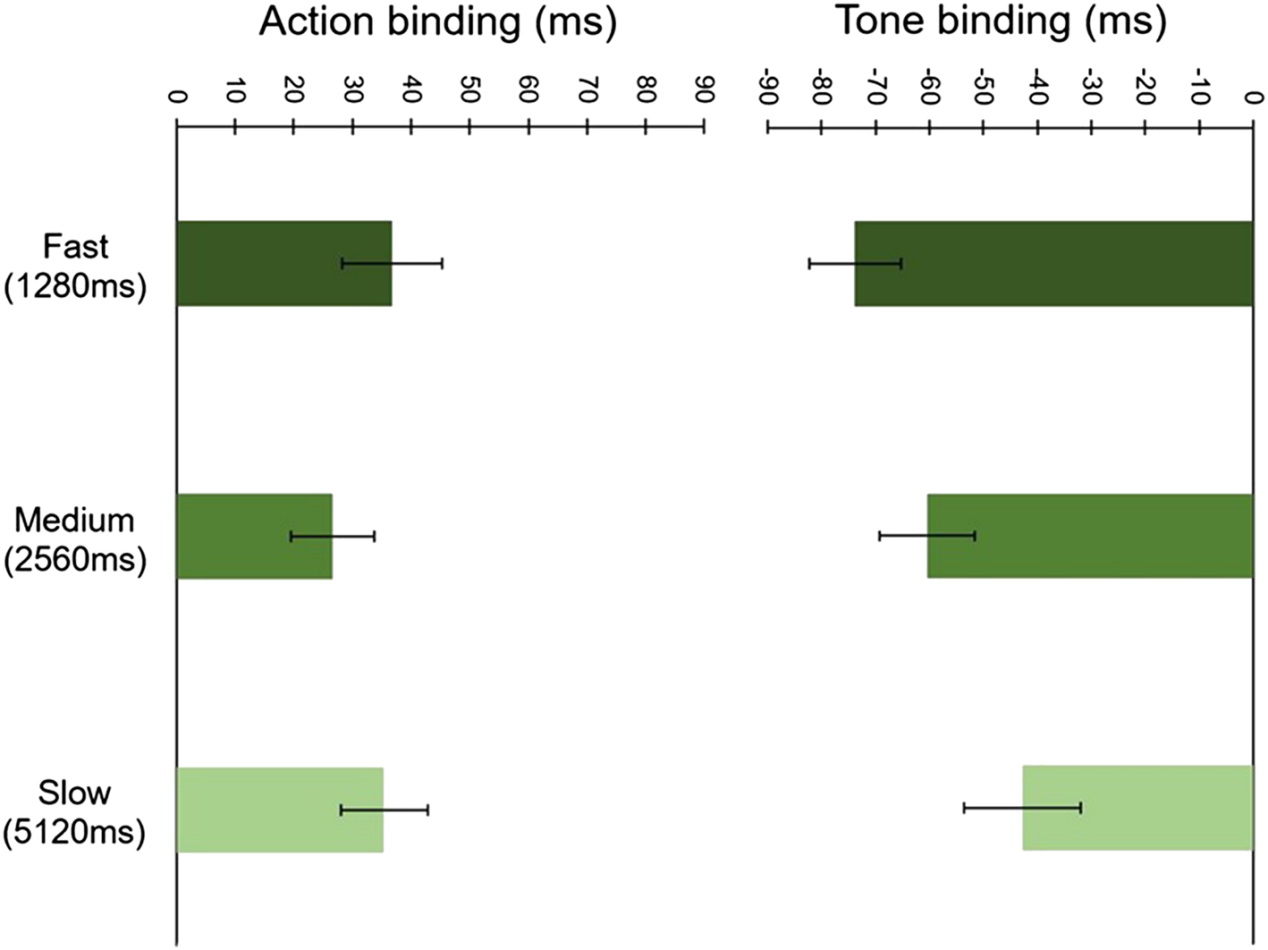
Mean baseline-corrected judgment errors across all clock speed conditions in Experiment 1. Error bars depict Cousineau (2005) within-subject CIs

The binding data for each clock speed and event type are shown in Fig. 2. We found no significant main effect of Speed, *F*(2, 76) = 2.26, *p* = 0.111, *ɳ*_p_^2^ = 0.056, and an expected significant main effect of Event, *F*(1, 38) = 55.66, *p* < 0.001, *ɳ*_p_^2^ = 0.594, reflecting that action and tone binding scores were significantly different to each other. Importantly, we found an ambiguous, non-significant Speed × Event interaction, *F*(2, 76) = 2.71, *p* = 0.073, *ɳ*_p_^2^ = 0.067. This suggests that Speed might have exhibited a weak effect on temporal binding.

To follow this interaction up, we conducted a repeated-measures ANOVA with Clock speed (1280 ms, 2560 ms, 5120 ms) as a within-subject factor and mean baseline-corrected action judgment error as a dependent variable. There was no significant main effect of Speed on Action binding scores, *F*(2, 76) = 0.93, *p* = 0.396, *ɳ*_p_^2^ = 0.024.

A subsequent repeated-measures ANOVA with Clock speed (1280 ms, 2560 ms, 5120 ms) as a within-subject factor and mean baseline-corrected tone judgment error as a dependent variable revealed a trend-level main effect of Speed on Tone binding, *F*(2, 76) = 3.12, *p* = 0.050, *ɳ*_p_^2^ = 0.076. Bonferroni-corrected pairwise comparisons examining the differences in tone binding scores as a function of clock speed showed that Tone binding ambiguously differed between the 1280 ms and 5120 ms conditions, *p* = 0.067, Cohen’s *d* = − 0.381, whereas it was non-significantly different between the 1280 ms and 2560 ms, *p* = 0.936, Cohen’s *d* = − 0.164, and the 2560 ms and 5120 ms conditions, *p* = 0.370, Cohen’s *d* = − 0.252. This suggests there is a trend for the change in speed from extremely slow (5120 ms) to extremely fast (1280 ms) to increase Tone binding, and it might be this effect that drives the inconclusive Speed x Event interaction reported above.

### Discussion

We manipulated the rotation speed of the Libet clock to see whether this would impact temporal binding similarly to how it reportedly did for basic timing judgments (Danquah et al., 2008). Although we observed robust binding effects, binding was not significantly altered by the speed manipulation. Nevertheless, there was weak evidence that temporal binding varied with clock speed—although action binding was not significantly increased by an increase in clock speed, tone binding did show this trend, being larger in the fastest as opposed to the slowest speed condition.

## Experiment 2

This experiment consisted of a direct replication of Experiment 1 to resolve the ambiguous Speed x Binding interaction. We expected an interaction between binding and clock speed driven by an increase in tone binding as a function of an increase in clock speed.

### Methods

#### Participants

We recruited 40 participants (*M*_age_ = 20 years, SD = 2.1, age range 18–26, 8 males) using the Goldsmiths Research Participation Scheme. We based our sample size on the same rationale as we did in Experiment 1. They were compensated with 7.5 course credits for approximately 1hr30mins of experimental time. We applied the same participant inclusion criteria as we did in Experiment 1. Participants provided written informed consent prior to participation and the experiment was approved by the Department Ethics Committee at Goldsmiths, University of London.

#### Materials, procedure and data analysis

The materials, procedure and data analysis protocol were identical to those of Experiment 1. We excluded 1.95% trials from the 1280 ms, 1.83% from the 2560 ms and 1.97% from the 5120 ms clock speed condition. No participants were excluded.

#### Statistical analyses

The Kolmogorov–Smirnov test did not render any significant results. We also did not find any abnormal skewness and kurtosis values, boxplots or histograms, so we proceeded forward using parametric statistics. Based on the ambiguous results of Experiment 1, we used simple contrasts when investigating one key effect (i.e. the difference in tone binding scores between the 1280 ms and 5120 ms clock speed condition) and Bonferroni-corrected post hoc tests when examining all other mean differences.

In the case of one analysis below, Mauchly’s Test revealed that the assumption of sphericity was violated, so we reported the Greenhouse–Geisser correction.

Due to the ambiguity present in Experiment 1, when investigating the interaction between clock speed and temporal binding as a whole and the effect of extreme speeds on tone binding, we supplemented frequentist analyses with four Bayes Factors (BFs). BFs represent a measure of the relative likelihood of one hypothesis relative to the other given the available data and can aid the interpretation of non-significant or ambiguous results (Dienes, 2014).

Following Dienes (2014), priors for the three BFs for the Speed x Binding interaction were derived by splitting the 3 (Clock speed: 1280 ms, 2560 ms, 5120 ms) × 2 (Event: Action binding, Tone binding) 2-degree of freedom effect from Experiment 1 into three subsidiary 2 (Clock speed) × 2 (Event) 1-degree of freedom effects involving all two-level speed comparisons (1280 vs 2560 ms; 2560 vs 5120 ms; and 1280 vs 5120 ms). In each case, we computed the mean difference in action and tone binding as a function of clock speed and summed these differences to form an omnibus clock speed effect on binding (collapsed across action and tone binding) for each clock speed difference. Finally, we computed the mean difference in tone binding between extreme clock speeds (1280 vs 5120 ms) in Experiment 1. These four values were subsequently used as priors for the assessment of different clock speed effects on binding in Experiment 2.

Each BF was calculated for the M and SE of the effect of interest in Experiment 2 using a half-normal distribution with 0 as the mean and the magnitude of the effect in Experiment 1 as the SD. Following convention, we interpreted BFs greater than 3 as providing moderate (or greater) evidence for the alternative hypothesis, less than 0.33 as moderate evidence for the null hypothesis, and between 0.33 and 3 as insensitive evidence (Dienes, 2011; Jeffreys, 1961).

### Results

Participants’ mean performance across all binding blocks and clock speed conditions is depicted in Table 2.

**Table 2:** Mean raw and baseline-corrected judgment errors as a function of clock speed

Rotation speed condition (ms/revolution)	Judged event	Mean raw judgment error (ms) (SD)	Mean shift from baseline (ms) (SD)
1280 ms	Action		
	Baseline	− 28.45 (55.55)	21.89 (38.87)
	Operant	− 6.56 (57.58)	
	Tone		
	Baseline	− 30.79 (52.63)	− 79.91 (92.08)
	Operant	− 110.71 (114.46)	
2560 ms	Action		
	Baseline	− 38.57 (70.07)	29.80 (38.66)
	Operant	− 8.77 (64.29)	
	Tone		
	Baseline	− 38.31 (62.10)	− 64.84 (100.33)
	Operant	− 103.15 (133.59)	
5120 ms	Action		
	Baseline	− 9.82 (77.43)	23.74 (58.18)
	Operant	13.91 (87.33)	
	Tone		
	Baseline	− 33.63 (73.19)	− 31.80 (133.47)
	Operant	− 65.43 (164.59)	

**Fig. 3:**
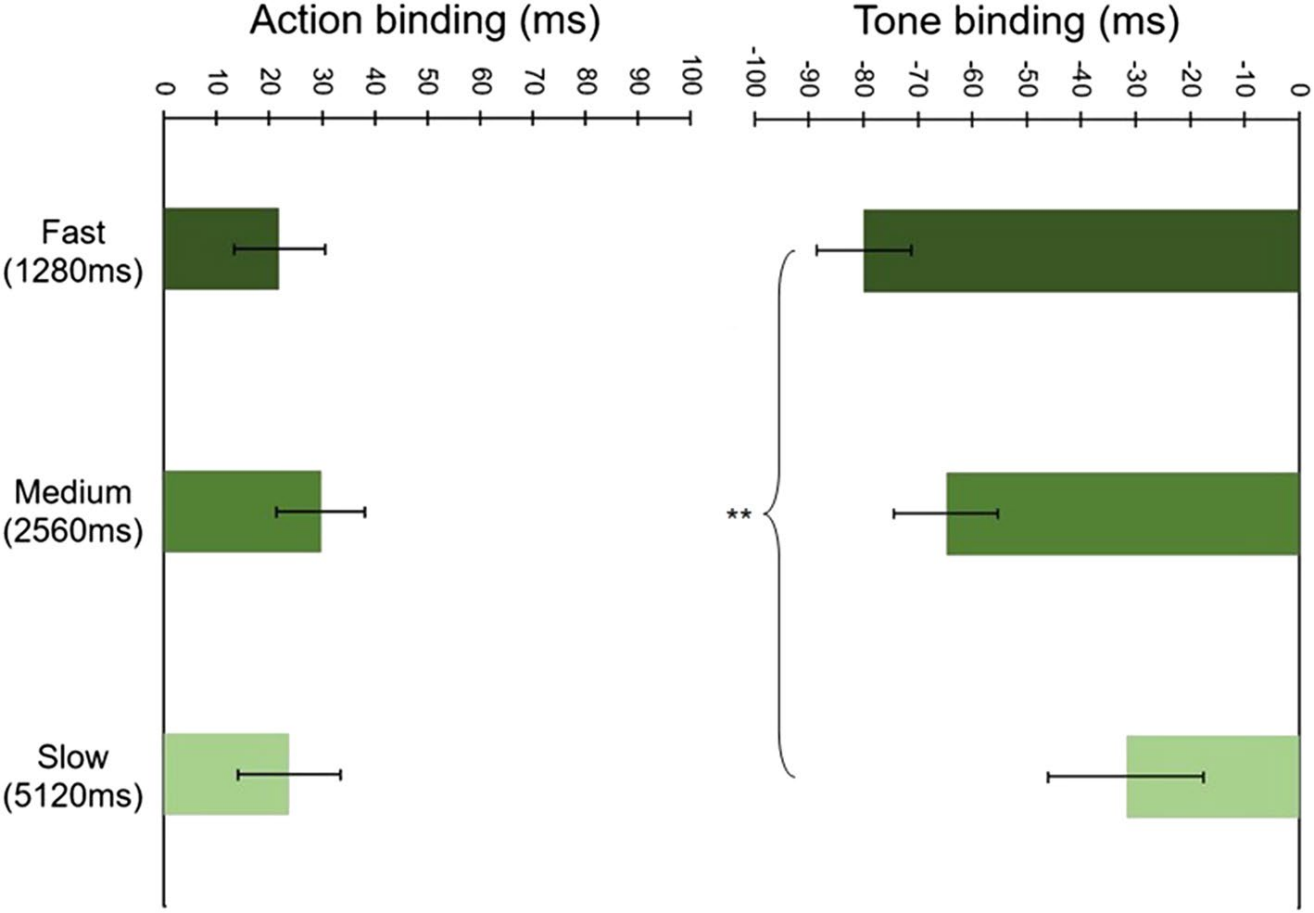
Mean baseline-corrected judgment errors across all speed conditions in Experiment 2. Error bars depict Cousineau (2005) within-subject CIs. **Indicates a significant difference between tone binding in the 1280 ms vs 5120 ms condition (*p* < 0.01)

In Fig. 3, participants’ mean action and tone binding scores are plotted as a function of clock speed. Unlike in Experiment 1, here, we found a significant main effect of Speed, *F*(2, 78) = 4.94, *p* = 0.010, *ɳ*_p_^2^ = 0.112. We again found a significant main effect of Event, *F*(1, 39) = 31.76, *p* < 0.001, *ɳ*_p_^2^ = 0.449. Crucially, this time we also observed a significant Speed x Event interaction, *F*(2, 78) = 4.29, *p* = 0.017, *ɳ*_p_^2^ = 0.099.

Two further repeated-measures ANOVAs examined the influence of clock speed on action and tone binding separately. As in Experiment 1, there was a non-significant main effect of Speed on Action binding, *F*(2, 78) = 0.47, *p* = 0.624, *ɳ*_p_^2^ = 0.012. In contrast, there was a significant main effect of Speed on Tone binding, *F*(2, 78) = 6.08, *p* = 0.003, *ɳ*_p_^2^ = 0.135, reflecting that tone binding was larger in the 1280 ms than in the 5120 ms condition, *F*(1, 39) = 10.18, *p* = 0.003, *ɳ*_p_^2^ = 0.207, thereby replicating this observation from Experiment 1. Moreover, inspection of Table 2 reveals that this increase in tone binding is driven by changes in perceived time of operant (rather than baseline) tones. Tone binding did not significantly differ between the other speed conditions, 1280 ms vs 2560 ms, *p* = 0.599, Cohen’s *d* = − 0.206, and 2560 ms vs 5120 ms, *p* = 0.114, Cohen’s *d* = − 0.340.

Computation of Bayes factors (BFs) for the Speed × Binding interaction revealed that the differences between the 1280 ms and 2560 ms, and the 2560 ms and 5120 ms clock speed conditions were insufficiently sensitive to corroborate the null hypothesis that these clock speeds do not affect binding, BF_[0, 23.47]_ = 0.77 and BF_[0, 8.85]_ = 2.47, respectively. In contrast, the difference between the 1280 ms and 5120 ms conditions seems to have affected binding, BF_[0, 32.32]_ = 10.31. Cumulatively, these results show a relationship between manipulations of the Libet clock speed and temporal binding that seems to be driven primarily by the effect of extreme speeds on binding.

The BF we calculated to probe the difference in tone binding between the extreme ends of the clock speed spectrum revealed strong evidence in favour of a difference in tone binding between the 1280 ms vs the 5120 ms speed condition, BF_[0, 30.9]_ = 53.36.

Together, these results suggest that clock speed affects temporal binding and that this effect is primarily driven by changes in tone binding, which, as predicted, increased in the fast relative to the slow clock speed condition.

### Discussion

This direct replication of Experiment 1 resolves the ambiguity present in Experiment 1. We found a significant interaction between clock speed and binding, suggesting that temporal binding is sensitive to manipulations of the measurement stimulus. More specifically, tone rather than action binding seems to be more sensitive to these manipulations—we found tone binding to increase linearly with an increase in clock speed and significantly differ between the extremities of the clock speed rotation. These results were strengthened by Bayes Factors, which brought support in favour of an interaction between speed and temporal binding as a whole and, most especially, in favour of greater tone binding in the 1280 ms as compared with the 5120 ms condition.

Taken together, the results of Experiment 1 and 2 suggest that clock speed has an effect on temporal binding. This result emphasises the need for consistency across studies in the setting of clock speed. This also implies that the expression of the binding effect itself is dependent, at least to some extent, on stimulus parameters, warranting a consideration of other stimulus parameters that might similarly impact on temporal binding.

## Experiment 3

In this experiment, we investigated the effect of clock markings on temporal binding. Unlike clock speed, this parameter varies considerably across studies (e.g. Demanet et al., 2013; Haggard et al., 2002; Lau et al., 2006; Libet et al., 1983; Trevena & Miller, 2002). We measured temporal binding with two different clock marker settings: the standard variant of the Libet clock marked at conventional intervals (5, 10, 15, etc.—condition 5′) and a variant where the clock was marked more granularly (1, 2, 3, etc.—condition 5′ + 1′).

### Methods

#### Participants

40 participants (*M*_age_ = 30.15 years, SD = 9.25, age range 18–61, 11 males) were recruited using existing databases and the Goldsmiths Research Participation Scheme. We elected our sample size in a similar way as we did in both experiments reported above. They were compensated with £7.5 or 5 course credits for approximately 1 h of experimental time. The final dataset contained 39 participants (*M*_age_ = 30.25 years, SD = 9.35, age range: 18–61, 10 males) after one participant exclusion was made (see “Data analysis”). We applied the same participant inclusion criteria as in Experiments 1 and 2. All participants provided written informed consent prior to participation and the experiment was approved by the Department Ethics Committee at Goldsmiths, University of London.

#### Materials

**Fig. 4:**
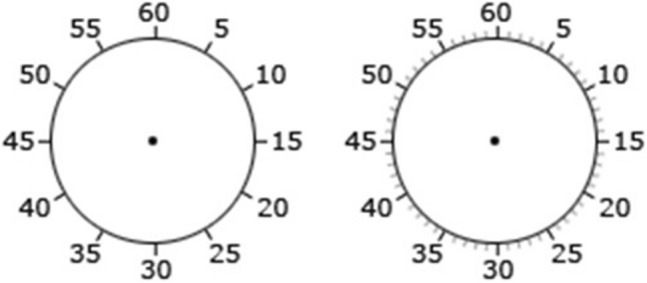
The two types of clock markings used across all experimental conditions in Experiment 3

Temporal binding was measured using the same Libet clock task as reported above. The clock rotated at the standard speed of 2560 ms per revolution, featured a 9 mm hand and measured 21 mm in diameter. Across all relevant conditions, the tone was presented at 1,000 Hz and lasted for 100 ms. Crucially, this time, we used two types of clock markings across two separate conditions (5′—the clock was marked in steps of five; 5′ + 1′—the clock was marked in steps of five and more granular steps of one; see Fig. 4).

#### Procedure

We used the same procedure as in Experiments 1 and 2 (see Fig. 1). During practice trials, participants performed the Libet task using a clock marked at conventional intervals (5, 10, 15, etc.).

#### Data analysis

This experiment used the same data analysis methods as the previous two experiments. This time, across all four binding blocks, 2.07% trials were excluded from the 5′ and 1.92% from the 5′ + 1′ clock markings conditions. One participant was excluded as an outlier on the basis of kurtosis scores and boxplot inspection.

#### Statistical analyses

We used the same type of frequentist analyses as we did in Experiments 1 and 2. That is, we conducted a factorial 2 × 2 repeated-measures ANOVA with Clock markings (5′, 5′ + 1′) and Event (Action binding, Tone binding) as within-subject factors and mean baseline-corrected judgment error as a dependent variable.

### Results

Participants’ mean action and tone timing scores across all four baseline and operant blocks, as well as their mean binding scores across both clock markings conditions, are shown in Table 3.

**Table 3:** Mean raw and baseline-corrected judgment errors as a function of two different types of clock markings

Clock markings condition	Judged event	Mean raw judgment error (ms) (SD)	Mean shift from baseline (ms) (SD)
5′	Action		
	Baseline	− 2.69 (89.34)	23.29 (41.81)
	Operant	20.60 (87.22)	
	Tone		
	Baseline	5.03 (75.54)	− 91.36 (113.65)
	Operant	− 86.33 (136.47)	
5′ + 1′	Action		
	Baseline	− 22.73 (82.24)	32.54 (35.85)
	Operant	9.80 (86.86)	
	Tone		
	Baseline	4.33 (71.36)	− 92.31 (114.57)
	Operant	− 87.98 (135.83)	

**Fig. 5:**
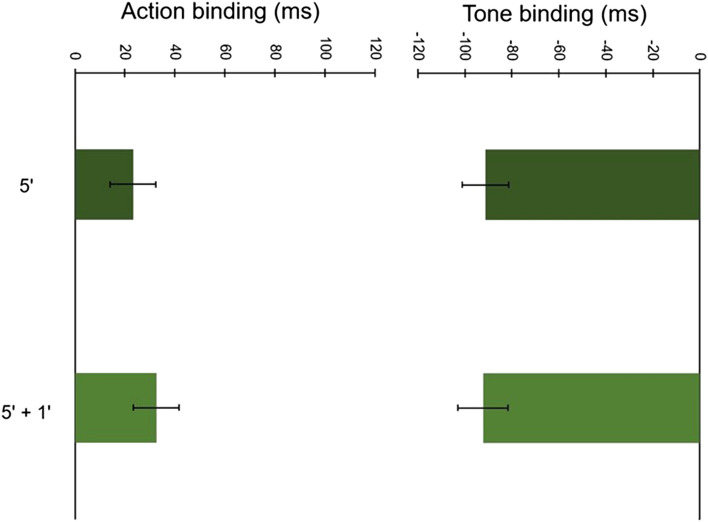
Mean baseline-corrected judgment errors across all clock markings conditions in Experiment 3. Error bars depict Cousineau (2005) within-subject CIs

A close inspection of Table 3 and Fig. 5 reveals a robust temporal binding effect that did not differ a great deal as a function of changes in clock markings. There was a non-significant effect of Clock markings, *F*(1, 38) = 0.35, *p* = 0.557, *ɳ*_p_^2^ = 0.009, and an expected significant main effect of Event, *F*(1, 38) = 51.01, *p* < 0.001, *ɳ*_p_^2^ = 0.573, reflecting the different directions of shift for Action binding (positive) and Tone binding (negative). The interaction between Clock markings and Event was non-significant, *F*(1, 38) = 0.57, *p* = 0.451, *ɳ*_p_^2^ = 0.015. These results suggest that manipulations of clock markings do not influence temporal binding.

### Discussion

In this experiment, we examined the impact of clock markings on temporal binding. Our results show that temporal binding did not significantly vary across markings conditions. These results further suggest that inconsistencies in clock markings across studies are unlikely to be problematic. Nevertheless, it is possible that our manipulations were not salient enough to impact temporal binding. To probe this possibility further, in our next experiment, we presented more extreme manipulations of clock markings.

## Experiment 4

**Fig. 6:**
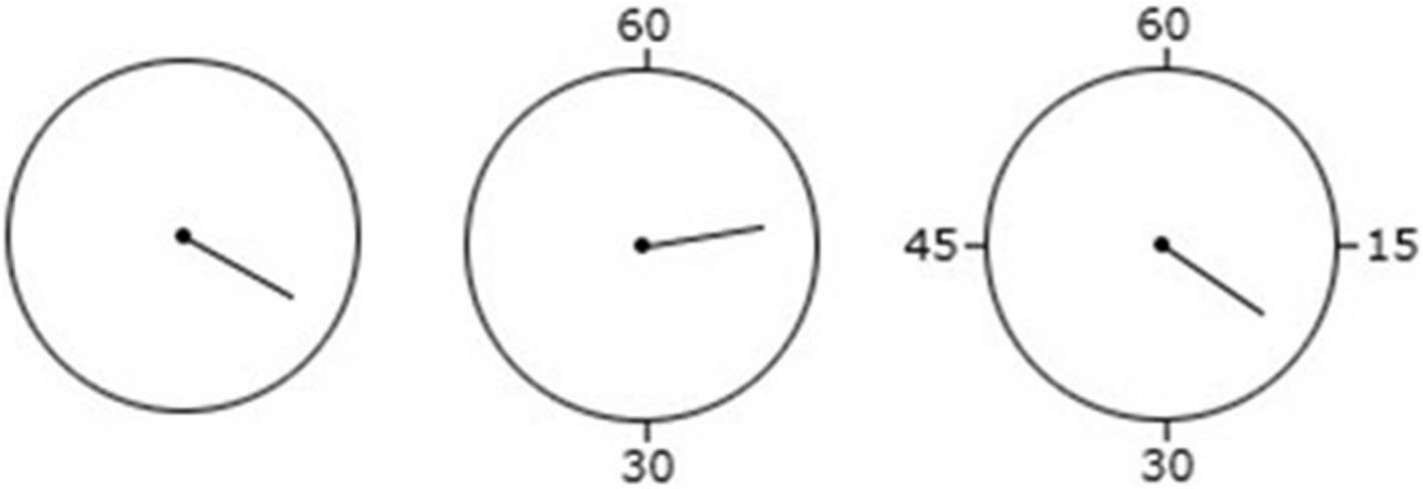
The three types of clock markings used across all experimental conditions in Experiment 4

This experiment examined the impact of different, more extreme clock markings configurations on temporal binding. To this end, participants completed the temporal binding task in three separate clock markings conditions: (1) no markings, (2) markings at 30′ intervals, and 3) markings at 15′ intervals (see Fig. 6).

### Methods

#### Participants

40 participants (*M*_age_ = 21.35 years, SD = 5.96, age range 18–36, 6 males) were recruited to participate in this study using existing databases and the Goldsmiths Research Participation Scheme. We based our sample size on criteria identical to those used in Experiments 1, 2 and 3. They were compensated with £10 or 7.5 course credits for approximately 1hr30mins of experimental time. The final dataset included 36 participants (*M*_age_ = 21.02 years, SD = 5.87, age range 18–36, 6 males) after participants exclusions were made. We used the same participant inclusion criteria as with the previous experiments above. Participants provided written informed consent prior to participation and the experiment was approved by the Department Ethics Committee at Goldsmiths, University of London.

#### Materials

Temporal binding was measured using the same Libet clock task as in Experiment 3. This time, we used three types of clock markings across three separate conditions (No markings—the clock was not marked at all, 30′—the clock was marked in steps of 30, 15′—the clock was marked in steps of 15; see Fig. 6).

#### Procedure

The procedure was identical to the one used in Experiment 3.

#### Data analysis

The data analysis protocol was identical to that of the previous experiments. Across all four binding blocks, we excluded 1.57% trials from the No markings, 1.82% from the 30′ and 1.85% from the 15′ condition. We also excluded four participants that were identified as outliers based on boxplot inspection.

#### Statistical analyses

The data were analysed using a 3 × 2 repeated-measures ANOVA with Clock markings (No markings, 30′, 15′) and Event (Action binding, Tone binding) as within-subject factors and mean baseline-corrected judgment error as a dependent variable.

### Results

Participants’ mean action and tone timing scores across all baseline and operant blocks, and their mean binding scores across all clock markings conditions are shown in Table 4.

**Table 4:** Mean raw and baseline-corrected judgment errors as a function of three different types of clock markings

Clock markings condition	Judged event	Mean raw judgment error (ms) (SD)	Mean shift from baseline (ms) (SD)
No markings	Action		
	Baseline	− 15.30 (45.71)	19.96 (42.29)
	Operant	4.65 (39.86)	
	Tone		
	Baseline	− 29.23 (55.12)	− 109.32 (113.28)
	Operant	− 138.56 (104.81)	
30′	Action		
	Baseline	− 18.94 (45.59)	19.98 (34.20)
	Operant	1.04 (41.29)	
	Tone		
	Baseline	− 25.27 (48.99)	− 120.20 (103.31)
	Operant	− 145.47 (106.49)	
15′	Action		
	Baseline	− 22.54 (54.23)	25.05 (37.50)
	Operant	2.51 (57.48)	
	Tone		
	Baseline	− 32.69 (56.55)	− 108.77 (101.71)
	Operant	− 141.47 (115.36)	

**Fig. 7:**
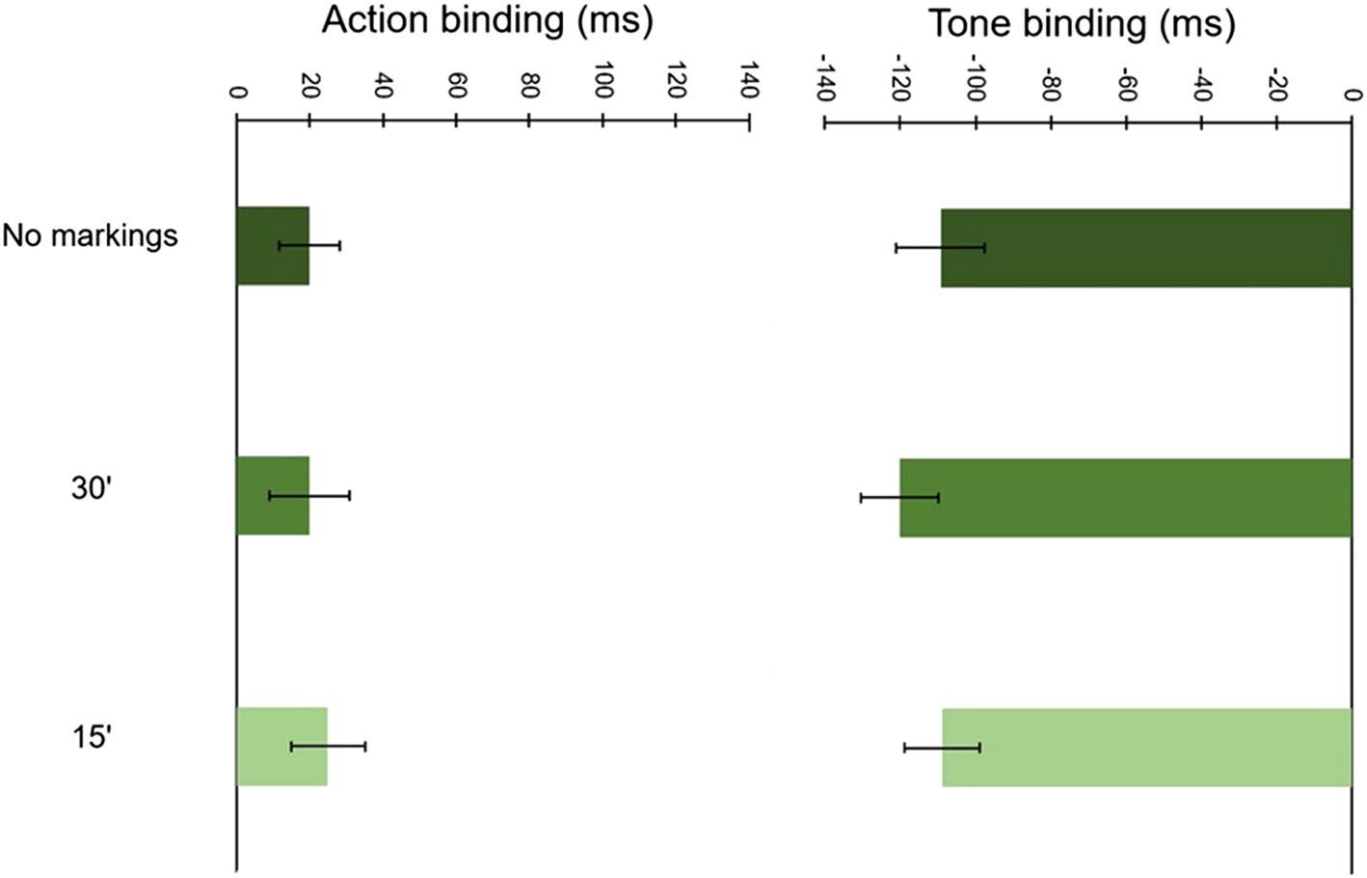
Mean baseline-corrected judgment errors across all clock markings conditions in Experiment 4. Error bars depict Cousineau (2005) within-subject CIs

As can be seen in Fig. 7, here was a non-significant main effect of Clock markings, *F*(2, 70) = 1.03, *p* = 0.361, *ɳ*_p_^2^ = 0.029, an expected significant main effect of Event, *F*(1, 35) = 58.34, *p* < 0.001, *ɳ*_p_^2^ = 0.625, and the interaction between Clock markings and Event was non-significant, *F*(2, 70) = 0.28, *p* = 0.756, *ɳ*_p_^2^ = 0.008. These results suggest that changes to clock markings do not have a significant effect on binding.

### Discussion

In this experiment, we investigated whether more extreme manipulations of clock markings would impact temporal binding. As in Experiment 3, we found that temporal binding was not significantly affected by this manipulation. Taken together, Experiments 3 and 4 suggest that inter-study variability in clock markings is unlikely to contribute to variability in the magnitude of binding across studies.

## Experiment 5

In our final experiment, we examined the impact of clock hand length on temporal binding. This stimulus parameter is not widely reported in studies. However, where it is reported it varies considerably across studies (e.g. Capozzi et al., 2016; Desantis et al., 2011; Engbert & Wohlschläger, 2006; Moore & Haggard, 2008; Takahata et al., 2012; Wenke et al., 2009). This variability, coupled with its under-reporting, highlights the need to examine the possible effect on temporal binding. To this end, we measured temporal binding in three clock hand length conditions (8 mm, 10 mm and 13 mm).

### Methods

#### Participants

Forty participants (*M*_age_ = 22.42 years, SD = 4.44, age range 18–35, 10 males) were recruited using existing databases and the Goldsmiths Research Participation Scheme. We chose our sample size according to the same principles present in all the experiments reported above. They were compensated with £10 or 7.5 course credits for approximately 1hr30mins of experimental time. The final dataset contained 39 participants (*M*_age_ = 22.38 years, SD = 4.49, age range 18–35, 9 males) after a single participant exclusion. We decided upon the participant inclusion criteria as we did in all experiments above. Participants provided written informed consent prior to participation and the experiment was approved by the Department Ethics Committee at Goldsmiths, University of London.

#### Materials

**Fig. 8:**
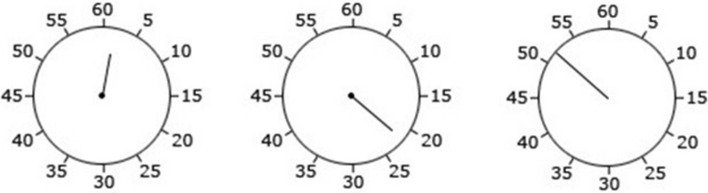
The three types of clock hand lengths used across all experimental conditions in Experiment 5—the clock hand was 8 mm (left), 10 mm (centre), 13 mm (right) long

Temporal binding was measured using the same Libet clock task as reported above. The clock rotated at the standard speed of 2560 ms per revolution, measured 21 mm in diameter and was marked at conventional intervals (5, 10, 15, etc.). Across all relevant conditions, the tone was presented at 1000 Hz and lasted for 100 ms. This time, we used three types of clock hand lengths across three separate conditions (8 mm, 10 mm or 13 mm; see Fig. 8).

#### Procedure

We used the same procedure as in all experiments above (see Fig. 1). During practice trials, participants were exposed to all three clock hand lengths.

#### Data analysis

The entire data analysis protocol used in this experiment was identical to the one used in our previous experiments. This time, across all four binding blocks, we excluded 1.77% from the 8 mm, 1.85% from the 10 mm and 2.07% trials from the 13 mm clock hand length conditions.

Due to abnormal kurtosis values and significant results rendered by the Kolmogorov–Smirnov test, we excluded a single subject from all subsequent analyses, who was also identified by boxplots as being an outlier.

#### Statistical analyses

We conducted a 3 × 2 repeated-measures ANOVA with Clock hand length (8 mm, 10 mm, 13 mm) and Event (Action binding, Tone binding) as within-subject factors and mean baseline-corrected judgment error as a dependent variable.

### Results

Participants’ mean action and tone timing scores across all baseline and operant blocks, and their mean binding scores across all clock hand length conditions are shown in Table 5.

**Table 5:** Mean raw and baseline-corrected judgment errors as a function of clock hand length

Clock hand length condition (mm)	Judged event	Mean raw judgment error (ms) (SD)	Mean shift from baseline (ms) (SD)
8	Action		
	Baseline	− 4.62 (51.55)	29.45 (39.72)
	Operant	24.83 (67.11)	
	Tone		
	Baseline	− 17.67 (48.97)	− 89.38 (92.57)
	Operant	− 107.05 (115.92)	
10	Action		
	Baseline	− 17.58 (56.82)	34.14 (56.30)
	Operant	16.56 (63.35)	
	Tone		
	Baseline	− 28.04 (57.73)	− 90.44 (100.84)
	Operant	− 118.48 (110.24)	
13	Action		
	Baseline	− 10.26 (43.66)	22.88 (34.98)
	Operant	12.61 (48.14)	
	Tone		
	Baseline	− 40.07 (40.10)	− 77.06 (95.10)
	Operant	− 117.14 (112.80)	

**Fig. 9:**
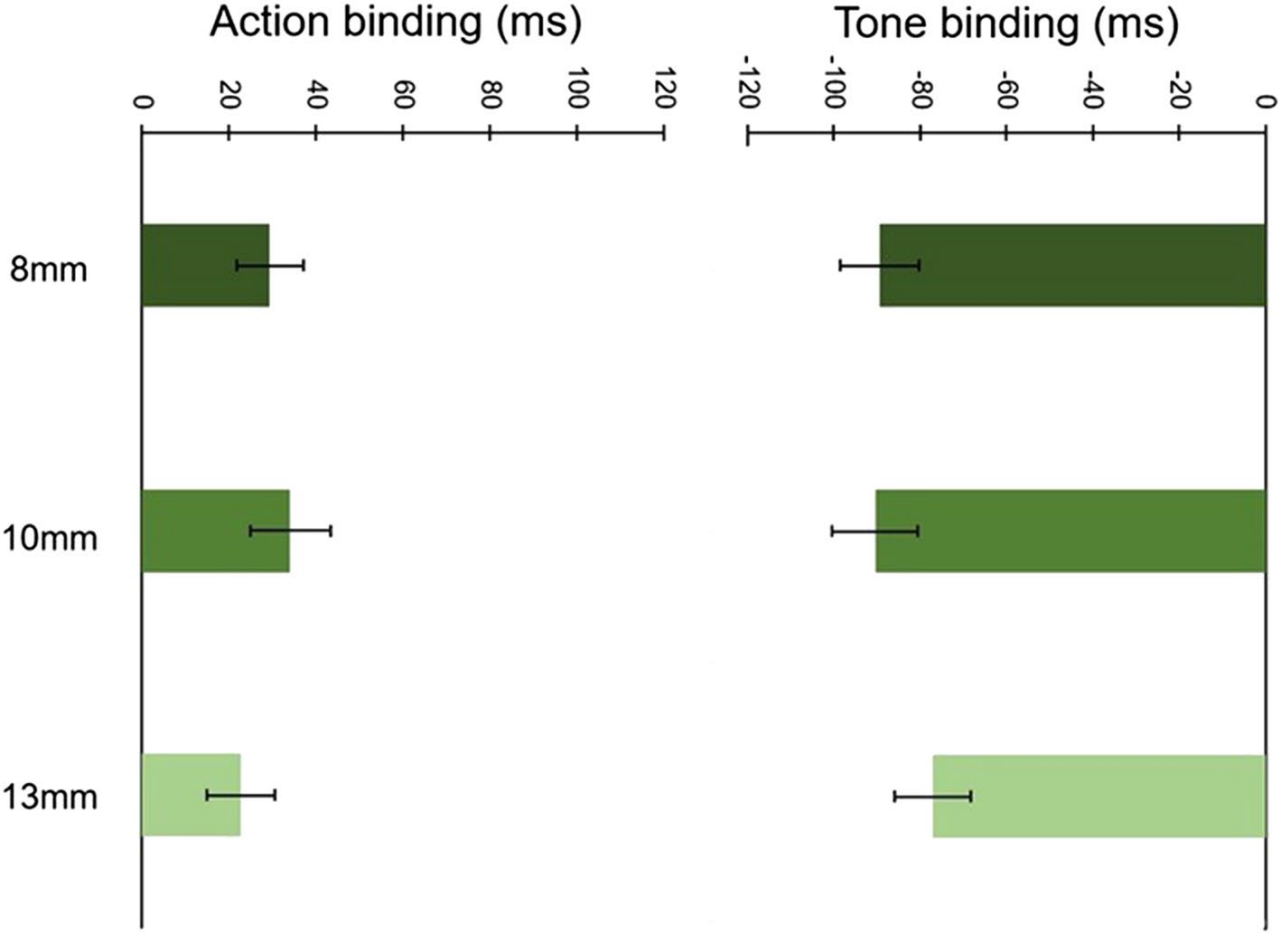
Mean baseline-corrected judgment errors across all clock hand length conditions. Error bars depict Cousineau (2005) within-subject CIs

A visual inspection of Table 5 and Fig. 9 reveals a robust temporal binding effect unaffected by manipulations of the clock hand length.

The main effect of Clock hand length was non-significant, *F*(2, 76) = 0.10, *p* = 0.899, *ɳ*_p_^2^ = 0.003, whereas the main effect of Event was significant, *F*(1, 38) = 64.07, *p* < 0.001, *ɳ*_p_^2^ = 0.628 (this showing that action and tone binding were significantly different from each other). The interaction between Clock hand length and Event was also non-significant, *F*(2, 76) = 2.14, *p* = 0.124, *ɳ*_p_^2^ = 0.053. This suggests that the length of the Libet clock hand does not influence temporal binding.

### Discussion

A manipulation of clock hand length did not seem to impact temporal binding. These results suggest that the inconsistencies in the literature regarding this parameter setting are not likely to be problematic.

## General discussion

In five experiments, we manipulated stimulus parameters of the Libet clock to examine the impact on temporal binding. We found that increasing clock speed impacts temporal binding by increasing tone binding. In contrast, manipulations of clock markings and length of the clock hand do not seem to affect either the action or tone component of temporal binding. These results have implications regarding the use of the Libet clock task in the measurement of temporal binding.

The stimulus parameters for the Libet clock vary considerably across studies. The principal finding of Experiments 1 and 2 is that intentional binding seems to be influenced by clock speed. This indicates that differential clock speeds across studies may contribute to inter-study heterogeneity in the magnitude of temporal binding. In turn, this result emphasises the need for consistency in this stimulus parameter, so that future investigations can accurately isolate the effect of their manipulations from that of other confounds on binding. This is important across experiments, especially if one is comparing effect sizes. It is also important within experiments if one is comparing temporal binding across groups.

Our other experiments examining clock markings and clock hand length suggest that changes to these parameters do *not* significantly influence the expression of temporal binding. Interestingly, these are parameters that often vary across experiments, but our results suggest that they are unlikely to be problematic. This is reassuring for those seeking to compare or aggregate findings from different experiments in which these parameters vary. It is also useful in terms of guiding the design of future studies—for example, when testing certain populations, it might be preferable to tweak some of these settings so as to reduce the perceptual burden of the task. Our findings show that this is unlikely to significantly alter temporal binding.

Although our findings have clear methodological implications, what is less clear is the underlying neurocognitive mechanisms, especially regarding the effect of clock speed on temporal binding. One possibility is that this result is linked to the effect that changing clock speed has on uncertainty. Eagleman and Holcombe (2002) propose that binding is the expression of a temporal contiguity prior for self-caused events, and that the existence of this prior, coupled with sensorimotor uncertainty, serves to bring actions and outcomes together in subjective experience. In line with this proposal, it is possible that an increase in the clock rotation speed increased participants’ uncertainty, thereby increasing (tone) binding—this would reflect an increased influence of the temporal contiguity prior on time estimates.

Alternatively, Waszak et al. (2012) pre-activation account of tone binding could provide a different explanation for our findings. According to this account, our actions pre-activate the representations associated with their outcomes. This pre-activation raises neural activity in the perceptual units representing these outcomes to some pedestal level, which makes them reach awareness faster. This, then, results in outcomes subjectively shifting towards the actions that caused them. One possibility is that increasing clock speeds increases the participants’ levels of arousal, which in turn could serve to further increase the mean level of activity in the perceptual units representing the anticipated effect. One consequence of this would be an increase in tone binding, which is what we observed. At present, we are unable to arbitrate between these competing explanations of our findings. However, this is something that could be more explicitly addressed in future experiments.

## Conclusions

In conclusion, the experiments presented in this paper found that changes in the Libet clock rotation speed seem to increase temporal binding, whereas manipulations of clock markings or the length of the clock hand do not seem to have a significant effect. Our results have various implications. First, they demonstrate that the Libet clock method constitutes a fairly robust temporal binding measure, which renders binding sensitive to some of its features, yet not to the extent that the effect is ever abolished altogether (at least not with the parameters we studied). This is important, taking into account that various other temporal binding measures have been developed in an attempt to tackle limitations of the Libet clock (see “Introduction”). Moreover, this also fits with Tanaka et al. (2019) view according to which the Libet clock method amplifies the magnitude of binding (in contrast to the interval estimation procedure, for example).

Second, our results highlight the importance of maintaining consistency with respect to the speed of the Libet clock across studies, and also the necessity of investigating the impact on binding of additional Libet clock features that vary across studies (e.g. the nature of the clock’s rotating object, its radius, or the Libet-style task instructions commonly used). Further research on these parameters is likely to provide greater clarity regarding the variables that modulate temporal binding.

## Data Availability

The datasets generated and analysed during the present studies are available at https://osf.io/j5x3a.

## Code availability

The code created and used during the present studies is available from the corresponding author on reasonable request.
